# Effect of Dietary Concentrate-to-Forage Ratios During the Cold Season on Slaughter Performance, Meat Quality, Rumen Fermentation and Gut Microbiota of Tibetan Sheep

**DOI:** 10.3390/ani14223305

**Published:** 2024-11-17

**Authors:** Shijia Wang, Wenhui Tang, Ting Jiang, Ru Wang, Ruoxi Zhang, Jingyu Ou, Qiangjun Wang, Xiao Cheng, Chunhuan Ren, Jiahong Chen, Yafeng Huang, Zijun Zhang

**Affiliations:** 1 College of Animal Science and Technology, Anhui Agricultural University, Hefei 230036, China; wangsj2024@ahau.edu.cn (S.W.); 23720297@stu.ahau.edu.cn (W.T.); jiangting2022@stu.ahau.edu.cn (T.J.); wangru3127@163.com (R.W.); zhangruoxi@stu.ahau.edu.cn (R.Z.); qq23720275@stu.ahau.edu.cn (J.O.); wangqiangjun@ahau.edu.cn (Q.W.); chengxiao@ahau.edu.cn (X.C.); renchunhuan@ahau.edu.cn (C.R.); chenjiahong@ahau.edu.cn (J.C.); huangyafeng@ahau.edu.cn (Y.H.); 2 Center of Agriculture Technology Cooperation and Promotion of Dingyuan County, Dingyuan 233200, China

**Keywords:** Tibetan sheep, carcass traits, meat quality, gut microbiota, correlation analysis

## Abstract

This study aimed to investigate the effects of different dietary concentrate-to-forage ratios on slaughter performance, meat quality, rumen fermentation, rumen microbiota and fecal microbiota in Tibetan sheep. A total of sixty male Tibetan sheep were equally allocated into three dietary groups based on concentrate-to-forage ratios, i.e., 30:70 (C30), 50:50 (C50), and 70:30 (C70). Compared with the C30 group, sheep fed the C70 diet resulted in a higher (*p* < 0.05) slaughter live weight (SLW), hot carcass weight (HCW), dressing percentage (DP), eye muscle area, average daily gain (ADG), and ruminal total volatile fatty acids concentration and propionate molar proportion and lower (*p* < 0.05) shear force and cooking loss of meat, and ruminal acetate molar proportion and acetate:propionate ratio. Sheep in the C50 group exhibited a higher (*p* < 0.05) SLW, HCW, ADG, and ruminal propionate molar proportion and lower (*p* < 0.05) shear force and cooking loss of meat, and ruminal acetate molar proportion and acetate: propionate ratio compared with the C30 group. In rumen fluid, the relative abundance of *Butyrivibrio* was lower (*p* = 0.031) in the C30 group, and that of *Ruminococcus* was higher (*p* = 0.003) in the C70 group compared with the C50 group. In feces, genus *Monoglobus* and *UCG_002* were the most abundant in the C30 group (*p* < 0.05), and the relative abundance of *Prevotella* was significantly higher in the C70 group than in other groups (*p* = 0.013). Correlation analysis revealed possible links between slaughter performance and meat quality and altered microbiota composition in the rumen and feces of Tibetan sheep. Overall, feeding a C70 diet resulted in superior carcass characteristics and meat quality in Tibetan sheep, thus laying a theoretical basis for the application of short-term remote feeding during the cold season.

## Simple Summary

Tibetan sheep are known for their high-quality meat and strong ability to adapt, but limited information is available on the effect of dietary concentrate-to-forage ratio on carcass traits, meat quality, rumen fermentation and gut microbiota of Tibetan sheep during the cold season. Therefore, this study investigated the impact of the different concentrate-to-forage ratios on slaughter performance, meat quality, rumen fermentation, and rumen and fecal microbial community composition. The results revealed that an increase in the dietary concentrate level from 30% to 70% positively impacted slaughter performance, tenderness, and juiciness of Tibetan sheep meat. Meat color was optimized with high acceptability by consumers and was observed at a dietary concentrate-to-forage ratio of 50:50. Correlation analysis revealed a strong correlation between specific gastrointestinal bacteria and slaughter performance as well as with meat quality. These findings offer insights into the effect of the diet on slaughter performance, meat quality, rumen microbiota, and fecal microbiota of Tibetan sheep, which may inform decisions regarding feeding strategies.

## 1. Introduction

Tibetan sheep (*Ovis aries*) are one of the most important livestock species living on the Qinghai-Tibetan Plateau (QTP), China, and a valuable genetic resource in China [1]. Tibetan mutton is prized for its rich aroma, fresh taste, tender texture, and high nutritional value due to its high protein, low cholesterol, and high vitamin and mineral contents [1]. These attributes make it a suitable choice among today’s consumers seeking high-quality and healthy meat [2]. However, due to the harsh natural environment in the QTP, the short growing season for forage is only approximately 120 days annually [1]. In particular, the quantity and quality of forage can decrease in the long cold season, which usually results in limited available nutrients when Tibetan sheep are fed exclusively on grazing [3]. This annual growth cycle results in lower productivity, food insecurity, and economic challenges for farmers. Sun et al. [4] demonstrated that short-term remote feeding is an effective alternative to natural grazing during the prolonged cold season, achieving desirable fattening outcomes in Tibetan sheep. A limited study by Zhang et al. [5] reported that transporting Tibetan sheep from Qinghai to Anhui, China, resulted in an average daily weight gain of 40–120 g/day from October to December, with blood parameters remaining within the normal range. Therefore, remote feeding could mitigate forage shortage and guarantee the short-term fattening of Tibetan sheep, preventing weight loss and meeting the peak demand for mutton during the cold season.

Carcass characteristics and meat quality in sheep are influenced by dietary factors, including the concentrate-to-forage (C:F) ratio and increasing the proportion of concentrate in ruminant diets is commonly used to meet the demand for high-quantity mutton during growth [6]. Previous studies have shown that higher proportions of concentrate in the diet resulted in faster and more efficient growth, as well as higher carcass weight [7,8], but may also impact physiological traits, including affecting the composition of the rumen and fecal microbiota [6,9]. Wang et al. [10] demonstrated that suitable dietary C:F ratios can provide balanced nutrition for ruminants, improved feed conversions and animal performance, as well as optimized rumen microbiota. Concentrate-based diets generally result in lower microbial complexity in the rumen and the feces compared with forage-based diets, largely due to the reduced variety of substrates [11]. However, as a unique breed on the QTP, a few studies on Tibetan sheep have only focused on the growth performance, rumen fermentation and bacterial diversity [12] or slaughter performance and meat quality [13] under different forage to concentrate ratios. To our knowledge, little comprehensive research has focused on the slaughter performance, meat quality, rumen fermentation, and rumen and fecal microbial community composition of Tibetan sheep, especially during short-term remote feeding in the cold season.

Given the above, the objectives of this study were to evaluate slaughter performance, meat quality, ruminal fermentation, and bacterial communities in the rumen and feces of Tibetan sheep-fed diets with three C:F ratios during the cold season. The findings discussed herein provide a theoretical basis and practical guidance for improving Tibetan sheep production through short-term remote feeding and inform future research on gut microbial metabolism in Tibetan sheep.

## 2. Materials and Methods

All experimental designs and protocols were approved by the Animal Care and Use Committee of Anhui Agricultural University, Hefei, China (approval No. AHAU2020023) and followed the institutional guidelines for animal research.

### 2.1. Animals, Diets and Experimental Design

Tibetan sheep were purchased from Henan Mongolian Autonomous County (Qinghai, China). The experiment was conducted from November 2023 to January 2024 at the Jianghuai Watershed Comprehensive Experiment Station of Anhui Agricultural University, Anhui Province, China. Sixty male Tibetan sheep (eight months old) with similar initial body weight (approximately 22.00 ± 0.30 kg) were randomly assigned to three experimental groups (*n* = 20) based on the feed C:F ratio (on a dry matter basis) in the diet: (i) 30:70 (C30 group); 50:50 (C50 group); and 70:30 (C70 group). All animals were vaccinated against internal and external parasites using an anthelmintic drug and were housed in individual pens (3.1 × 1.0 m) with five animals per pen in the same sheep shed. Sheep were provided free access to water and fed twice daily at 08:00 and 17:00 throughout the duration of the experiment, which lasted for 65 days following a 15-day adaptation period. No heating or humidity reduction measures were used during the fattening period. In the feeding area during the growing period (November 2023–January 2024), the minimum, maximum, and total average daily air temperatures were −6.4, 20.2 and 4.9 °C and the minimum, maximum, and total mean daily relative humidity was 39.0%, 99.0%, and 74.67%, respectively. The ingredients of the diets and nutritional composition are presented in Table 1.

### 2.2. Animal Slaughter and Carcass Characteristics

At the end of the experiment, five Tibetan sheep from each treatment group were randomly selected and individually weighed after fasting for 24 h and water restriction for 8 h to determine slaughter live weight (SLW). These animals were then slaughtered by professionals using the Halal method [14]. Average daily gain (ADG) was calculated as total weight gain divided by the number of days following the method described by Liu et al. [12] Hot carcass weight (HCW) was determined immediately after slaughter, and dressing percentage (DP) was calculated immediately after according to the method proposed by Zhang et al. [15] The grade rule (GR) value was measured using a Vernier caliper to determine tissue thickness between the 12th and 13th ribs at the 11 cm midline of the dorsal spine [16]. Eye muscle area (EMA) was determined by measuring the cross-sectional area of the *longissimus dorsi* muscle between the 12th and 13th ribs [14]. The right side of the *longissimus dorsi* muscle was sampled for subsequent meat quality analysis.

### 2.3. Meat Quality Determination

The *longissimus dorsi* muscle was collected to assess various meat quality attributes, including color, pH, drip loss (DL), cooking loss (CL), and shear force. Lightness (L*), redness (a*), and yellowness (b*) values were measured using a Minolta chromameter (ADCI-WSI, Beijing Chentaike Instrument Co., Ltd., Beijing, China). The pH of the muscle tissue was determined after 45 min (pH_45min_) and again at 24 h (pH_24h_) using a pH meter (FE20, Mettler Toledo International Inc., Stockholm, Sweden). The DL was determined as the percentage of water loss after storage at 4 °C for 24 h relative to the initial sample weight (1 cm × 1 cm × 2 cm) [17]. Samples were placed in polyethylene bags and cooked in a water bath at 75 °C for 1 h and cooking loss was recorded as the difference in weight before and after cooking. Shear force was measured parallel to the orientation of muscle fibers using a Warner-Bratzler machine (TMS-Pro, Food Technology Corp., Sterling, VA, USA). All experiments were performed in triplicate to calculate average values.

### 2.4. Rumen Fluid and Fecal Sampling and Ruminal Fermentation Analysis

After slaughter, rumen fluid samples were collected and immediately filtered into sterile freezing tubes using four layers of sterile gauze. Fecal samples were obtained from the rectum and placed in 10 mL sterile freezing tubes. Both rumen fluid and fecal samples were immediately frozen in liquid nitrogen and stored at −80 °C for subsequent analysis.

The analysis of volatile fatty acids (VFA, i.e., acetate, propionate, butyrate, isobutyrate, valerate and isovalerate) was performed on a capillary gas chromatograph (C-2010 Plus, Shimadzu, Kyoto, Japan) equipped with a capillary column (30 mm × 0.32 mm × 0.25 mm) as described by Eikanger et al. [18] Briefly, samples were thawed on ice and centrifuged at 20,000× *g* for 15 min. Then, 9.5 mL of supernatant was transferred into a new tube, enriched with 0.5 mL of 98% internal standard (formic acid) and centrifuged again. Subsequently, VFA concentrations were determined from the clear supernatant.

### 2.5. DNA Extraction, Microbiome Sequencing, and Analysis

High-throughput sequencing was performed at BioMarker Technologies Co., Ltd. (Beijing, China). Total genomic DNA was extracted using a Magnetic Soil and Stool DNA kit (Tiangen Biochemical Technology, Beijing, China) according to the manufacturer’s instructions. The DNA purity and concentration were verified by 1.8% agarose gel electrophoresis and with a Thermo NanoDrop 2000 spectrophotometer (Thermo Fisher Scientific, Waltham, MA, USA). The V3–V4 hypervariable regions of the prokaryotic 16S rRNA gene were amplified using the primers 338F (5′-ACTCCTACGGGAGGCAGCA-3′) and 806R (5′-GGACTACHVGGGTWTCTAAT-3′) [19]. The PCR amplification was performed in a reaction mixture containing 50 ng of template DNA, 0.3 μL of each primer, 5 μL of KOD FX Neo Buffer, 2 μL of 2 mM dNTPs, 0.2 μL KOD FX Neo and PCR-grade water to a total final volume of 10 µL. PCR conditions were as follows: initial denaturation at 95 °C for 5 min, followed by 25 cycles of denaturation at 95 °C for 10 s, annealing at 50 °C for 30 s, and extension at 72 °C for 40 s, and then final extension at 72 °C for 10 min. The PCR amplification products were evaluated using 1.8% agarose gel electrophoresis and purified using an E.Z.N.A. Cycle-Pure Kit (OMEGA, Norcross, GA, USA) and Monarch^®^ DNA Gel Extraction Kit (NEB, Ipswich, MA, USA). Amplicon pools were sequenced in an Illumina Novaseq 6000 platform (Illumina Inc., San Diego, CA, USA) using paired-end sequencing.

Raw data were processed using FLASH (version 1.2.11) and UCHIME (version 8.1). Sequences were classified into operational taxonomic units (OTUs) using a 97% threshold using USEARCH (version 10.0) and QIIME2 software platform (v2023.9), and chimeric sequences were identified and deleted. R software (3.2.1, CRAN, Vienna, Austria) was used for plotting, including the Venn diagram, PCoA, ANOSIM, and heatmap. Alpha diversity based on Chao 1, Good’s coverage, Shannon index, and Simpson’s index was calculated using Mothur (version 1.30.0) [20]. Principal coordinates analysis (PCoA) based on weighted UniFrac distances was conducted to compare samples, and a distance-based matrix analysis was performed to evaluate differences among samples. Sequence alignment was performed using the QIIME2 plugin feature classifier and the Silva database Release 138.

### 2.6. Statistical Analysis

Statistical analyses of slaughter performance, meat quality attributes and ruminal fermentation were performed using a general linear model routine in SPSS software (version 25.0, SPSS Inc., Chicago, IL, USA) and then evaluated using one-way ANOVA. Differences between treatments were evaluated using Duncan’s multiple range tests, with statistical significance set at *p* values < 0.05. Kruskal–Wallis test and Wilcoxon test were used to analyze the differences between groups at phylum and genus levels. *p*-Values were adjusted for multiple comparisons using the Benjamini–Hochberg correction method, and biomarkers with statistically significant differences were identified using LEfSe analysis considering a threshold of LDA ≥ 3.0 and *p* < 0.05 as screening criteria. Pearson correlation analysis was used to analyze relationships between rumen or fecal relative bacterial abundance, carcass performance, and meat quality, with significant correlations set as *p* < 0.05.

## 3. Results

### 3.1. Slaughter Performance

Table 2 shows the results of the effects of dietary C:F ratio on slaughter performance. The SLW and ADG are significantly lower (*p* < 0.001) in the C30 group than in the C50 or C70 group, with no difference between the C50 and C70 groups (*p* > 0.05). The HCW of the C50 group was higher (*p* < 0.001) than that of the C30 group; however, it was lower than that of the C70 group. The DP was lower (*p* = 0.016) in the C30 group than in the C70 group, with the C50 group being intermediate. The EMA was higher (*p* = 0.004) in the C70 group than in the other two groups. The grade rule averaged 0.85 cm and did not differ among the three treatment groups (*p* > 0.05).

### 3.2. Meat Quality Attributes

No significant differences (*p* > 0.05) were observed for pH_45min_, pH_24h_, L*, and a*, with average values of 6.41 ± 0.06, 5.50 ± 0.02, 33.90 ± 0.51, and 9.77 ± 0.23, respectively (Table 3). The shear force, cooking loss, and yellowness of meat are the highest (*p* < 0.001) for the C30 group and lowest (*p* < 0.05) for the C70 group, with the C50 group being intermediate. The drip loss of meat is higher (*p* < 0.001) in the C30 and C50 groups compared with the C70 group.

### 3.3. Rumen Fermentation Parameters

Total VFA concentration is significantly higher (*p* = 0.027) in the C70 group compared with the C30 and C50 groups, with no differences observed between the C30 and C50 groups (Table 4). The molar proportion of acetate and acetate:propionate are highest (*p* < 0.001) in the C30 group, intermediate in the C50 group and the lowest in the C70 group. The molar proportion of propionate was lower (*p* < 0.001) in the C50 group compared with the C70 group, but it was higher than that of the C30 group. The molar proportion of isobutyrate was lower (*p* = 0.049) in the C70 group compared with the C50 group, with the C30 group showing intermediate values. The molar proportions of butyrate, valerate, and isovalerate were similar (*p* > 0.05) among the three groups and averaged 10.78 ± 0.83, 3.95 ± 0.18, and 7.26 ± 0.22, respectively.

### 3.4. Rumen and Fecal Bacterial Diversity

**Figure 1:**
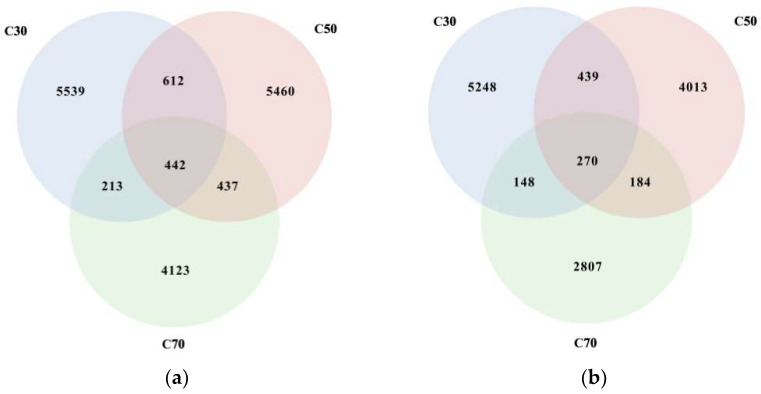
Effect of dietary concentrate-to-forage ratios on the Venn analysis of operational taxonomic units from the ruminal (**a**) and fecal (**b**) microbiota of Tibetan sheep (5 animals in each group). Abbreviations: C30 = 30:70 concentrate: forage; C50 = 50:50 concentrate: forage; C70 = 70:30 concentrate: forage.

The Venn diagram analysis revealed 5539, 5460, and 4123 unique OTUs in the rumen fluid (Figure 1a) and 5248, 4013, and 2807 unique OTUs in the feces (Figure 1b) of Tibetan sheep in the C30, C50, and C70 groups, respectively.

Alpha-diversity indicators of the rumen fluid and feces are presented in Table 5. The alpha-diversity and beta-diversity of microorganisms in the rumen and feces were evaluated to explore the differences in microbial composition among the three groups. In rumen fluid, Chao1 and Shannon indices were significantly lower (*p* < 0.05) in the C70 group compared with the C30 group, with the C50 group showing intermediate values. In feces, the Shannon index was the highest in the C30 group and the lowest in the C70 group (*p* = 0.039), while the PD_whole_tree was the highest in the C50 group and the lowest in the C70 group (*p* = 0.007). No significant differences (*p* > 0.05) were observed for the Simpson index, PD_whole_tree, or coverage in rumen fluid, nor for Chao1 and Simpson indices and coverage in feces across the three treatment groups. The above results indicate that a higher C:F ratio reduces bacterial richness and diversity in the rumen and reduces bacterial diversity in the feces of Tibetan sheep. The coverage across all samples exceeded 99.98%, suggesting that the sequencing depth was adequate for accurately characterizing the microbial composition of each sample.

**Figure 2:**
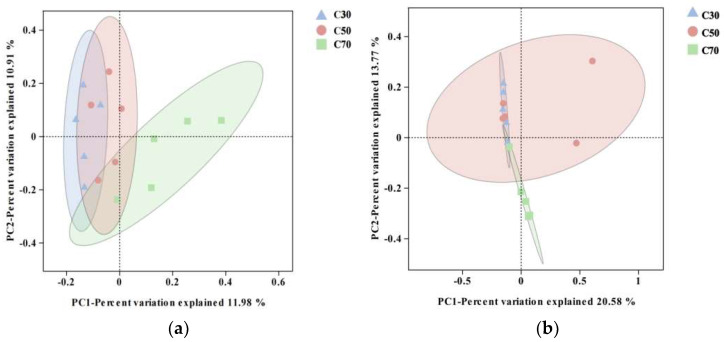
Effect of dietary concentrate-to-forage ratios on principal coordinate (PCoA) analysis of the microorganisms of the rumen (**a**) and feces (**b**) of Tibetan sheep (5 animals in each group). Abbreviations: C30 = 30:70 concentrate: forage; C50 = 50:50 concentrate: forage; C70 = 70:30 concentrate: forage.

The PCoA of rumen fluid samples showed that PCoA1 and PCoA2 accounted for 11.98% and 10.91% of the variance in the total microbial community structure, respectively (Figure 2a). In fecal samples, PCoA1 and PCoA2 accounted for 20.58% and 13.77% of the variation, respectively (Figure 2b). The ANOSIM results of rumen fluid samples showed significant differences in bacterial community structure between the C30 and C70 groups (*R* = 0.524, *p* = 0.007) and between C50 and C70 groups (*R* = 0.260, *p* = 0.020; Supplementary Table S1). In fecal samples, the distinction in the bacterial community revealed that significant differences were observed between the C30 and C70 groups (*R* = 0.660, *p* = 0.007) and between the C50 and C70 groups (*R* = 0.268, *p* = 0.028). The above results suggest that the dietary C:F ratio could substantially influence the diversity and abundance of microbiota in the rumen and feces of Tibetan sheep.

### 3.5. Rumen and Fecal Bacterial Community Structure

**Figure 3:**
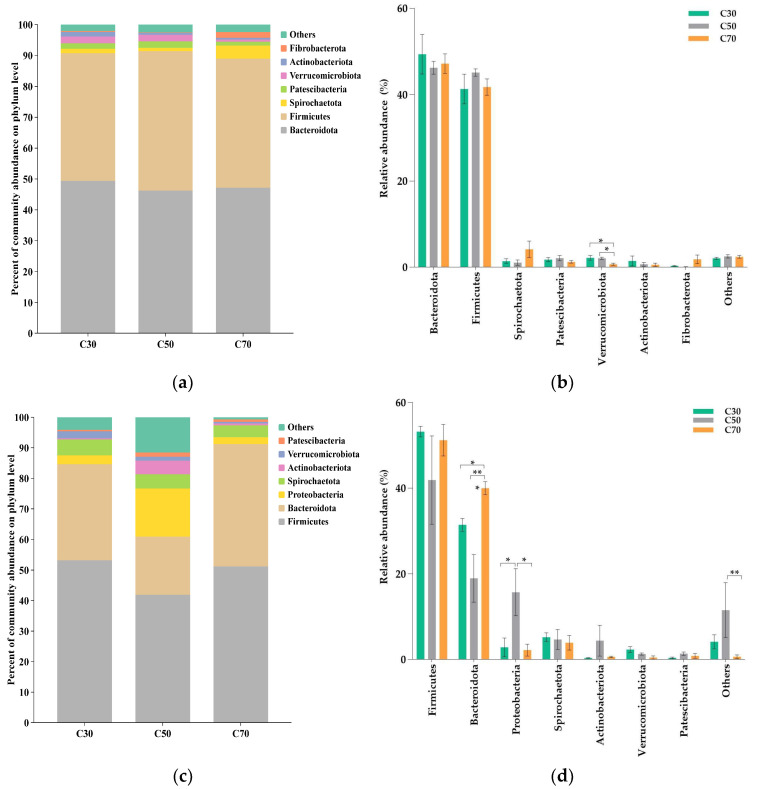
Effect of dietary concentrate-to-forage ratios on relative abundance of ruminal (**a**,**b**) and fecal (**c**,**d**) microbiome at the phylum level of Tibetan sheep (5 animals in each group). Abbreviations: C30 = 30:70 concentrate: forage; C50 = 50:50 concentrate: forage; C70 = 70:30 concentrate: forage; * 0.01 < *p* < 0.05, ** 0.001 < *p* < 0.01.

The relative abundances of the top seven phyla in rumen fluid and feces are illustrated in Figure 3. In rumen fluid, the most dominant phyla were similar (*p* > 0.05) across the three experimental groups, i.e., *Bacteroidota* (46.24–49.38%) and *Firmicutes* (41.33–45.15%; Figure 3a). The relative abundance of *Verrucomicrobiota* was significantly higher (*p* = 0.018) in the C30 and C50 groups compared with the C70 group (Figure 3b). No significant differences (*p* > 0.05) were observed for the relative abundances of the other phyla across the three treatment groups. In feces, *Firmicutes* (41.92–53.23%) and *Bacteroidota* (18.99–40.02%) were the two most dominant phyla (Figure 3c), with *Bacteroidota* being significantly more abundant (*p =* 0.004) in the C70 group compared with the C30 and C50 groups (Figure 3d). The relative abundance of *Proteobacteria* was significantly higher (*p =* 0.048) in the C50 group compared with the C30 and C70 groups. No significant differences were observed for the relative abundances of the other five phyla among the three treatment groups (*p* > 0.05).

**Figure 4:**
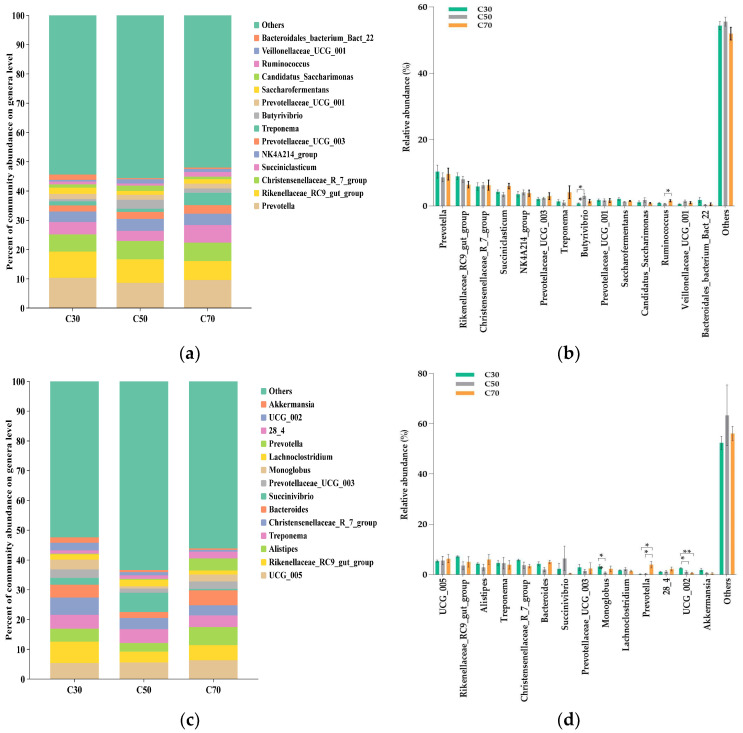
Effect of dietary concentrate-to-forage ratios on relative abundance of ruminal (**a**,**b**) and fecal (**c**,**d**) microbiome at the genus levels of Tibetan sheep (5 animals in each group). Abbreviations: C30 = 30:70 concentrate: forage; C50 = 50:50 concentrate: forage; C70 = 70:30 concentrate: forage; * 0.01 < *p* < 0.05, ** 0.001 < *p* < 0.01.

The relative abundances of the top 14 bacterial genera in rumen fluid (a) and feces (b) are shown in Figure 4. In rumen fluid, the relative abundance of *Butyrivibrio* was significantly lower (*p* = 0.031) in the C30 group, and that of *Ruminococcus* was higher (*p* = 0.003) in the C70 group compared with the C50 group (Figure 4a,b). The relative abundances of twelve other abundant genera were similar across treatments (*p* > 0.05). In feces, the relative abundance of *Monoglobus* was considerably lower (*p* = 0.018) in the C50 group than in the C30 group, with the C70 group showing an intermediate value (Figure 4c,d). Compared with the C30 group, the relative abundance of *Prevotella* was significantly higher (*p* = 0.031) in the C70 group, and that of *UCG_002* was lower (*p* = 0.013) in the C50 and C70 groups. The relative abundances of nine other abundant genera were similar across treatments (*p* > 0.05).

**Figure 5:**
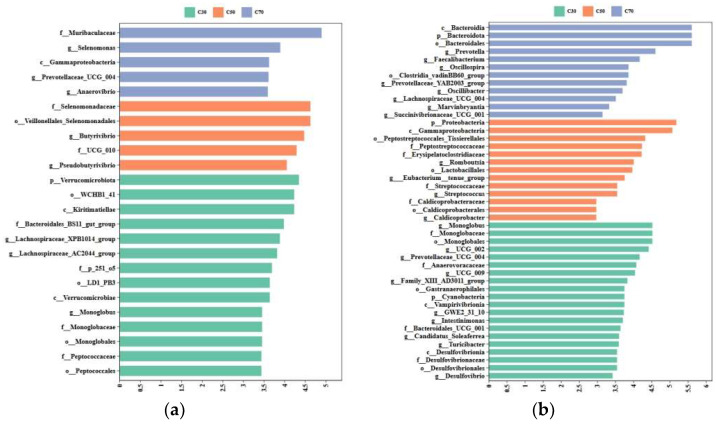
Effect of dietary concentrate-to-forage ratios on ruminal (**a**) and fecal (**b**) microbial composition using the linear discriminant analysis effect size analysis (LDA > 3 and *p* < 0.05) in Tibetan sheep (5 animals in each group). Abbreviations: C30 = 30:70 concentrate: forage; C50 = 50:50 concentrate: forage; C70 = 70:30 concentrate: forage.

Based on an LDA of 3.0, 16 genera were found to be significantly enriched among the three treatment groups (Figure 5). In rumen fluid, the genera *Lachnospiraceae_AC2044_group*, *Lachnospiraceae_XPB1014_group*, and *Monoglobus* were significantly enriched (*p* < 0.05) in the C30 group (Figure 5a). *Butyrivibrio* and *Pseudobutyrivibrio* were significantly enriched (*p* < 0.05) in the C50 group, and *Selenomonas*, *Anaerovibrio*, and *Prevotellaceae_UCG_004* were significantly enriched (*p* < 0.05) in the C70 group. 

In feces, the genera *Monoglobus*, *UCG_002*, *Prevotellaceae_UCG_004*, *UCG_009*, *GWE2_31_10, Family_XIII_AD3011_group*, *Intestinimonas*, *Turicibacter*, *Candidatus_Soleaferrea*, and *Desulfovibrio* were significantly enriched (*p* < 0.05) in the C30 group (Figure 5b); *Romboutsia*, *Eubacterium__tenue_group*, *Streptococcus*, and *Caldicoprobacter* were significantly enriched (*p* < 0.05) in the C50 group; and *Prevotella*, *Faecalibacterium*, *Prevotellaceae_YAB2003_group*, *Oscillospira*, *Lachnospiraceae_UCG_004, Oscillibacter*, *Succinivibrionaceae_UCG_00*1, and *Marvinbryantia* were significantly enriched (*p* < 0.05) in the C70 group.

### 3.6. Correlation Analysis

**Figure 6:**
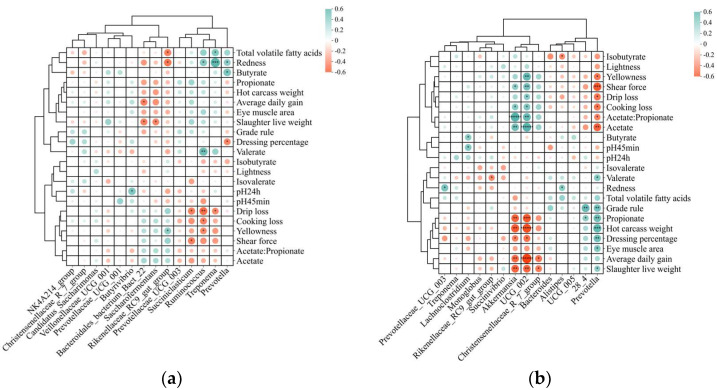
Pearson correlation coefficients between ruminal (**a**) and fecal (**b**) microbiota composition at the genus level and slaughter performance and meat quality (*n* = 15) of Tibetan sheep. * *p* < 0.05, ** *p* < 0.01, *** *p* < 0.001, ****** *p* < 0.000001.

To elucidate the relationship between rumen or fecal microbial composition and slaughter performance and meat quality in Tibetan sheep, a Pearson correlation analysis between these variables was carried out, and the significantly correlated substances (*p* < 0.05) are presented in Figure 6. In rumen fluid, *Bacteroidales_ bacterium_Bact_22* was found to be negatively correlated with SLW (*p* = 0.037, r = −0.559) and ADG (*p* = 0.031, r = −0.558; Figure 6a), while *Saccharofermentans* was negatively correlated with SLW (*p* = 0.037, r = −0.543). *Succiniclasticum* was negatively correlated with shear force (*p* = 0.026, r = −0.572) and DL (*p* = 0.026, r = −0.572). *Ruminococcus* was negatively correlated with DL (*p* = 0.009, r = −0.645), CL (*p* = 0.03, r = −0.559), b* value (*p* = 0.043, r = −0.529), and positively correlated with a* value (*p* = 0.025, r = 0.576) and molar proportion of valerate (*p* = 0.005, r = 0.685). *Treponema* was negatively correlated with DL (*p* = 0.043, r = −0.527) and positively correlated with a* value (*p* = 0.001, r = 0.773) and total VFA concentration (*p* = 0.023, r = 0.582). *Butyrivibrio* was positively correlated with pH_24h_ (*p* = 0.018, r = 0.60). *Prevotella* was positively correlated with a* value (*p* = 0.037, r = 0.542) and molar proportion of butyrate (*p* = 0.037, r = 0.614) and negatively correlated with DP (*p* = 0.039, r = −0.537). *Rikenellaceae_RC9_gut_group* was negatively correlated with a* value (*p* = 0.016, r = −0.610) and total VFA concentration (*p* = 0.027, r = −0.568), and positively correlated with b* value (*p* = 0.039, r = 0.537).

In feces, *Christensenellaceae_R_7_group* was negatively correlated with SLW (*p* = 0.021, r = −0.589) and ADG (*p* = 0.014, r = −0.618; Figure 6b). *Prevotella* was positively correlated with SLW (*p* = 0.042, r = 0.530), HCW (*p* = 0.001, r = 0.747), DP (*p* = 0.006, r = 0.672), EMA (*p* = 0.042, r = 0.530), grade rule (*p* = 0.009, r = 0.651), and molar proportion of propionate (*p* = 0.004, r = 0.691) and valerate (*p* = 0.023, r = 0.583), and negatively correlated with shear force (*p* = 0.001, r = −0.747), CL (*p* = 0.015, r = −0.614), DL (*p* = 0.013, r = −0.622), b* value (*p* = 0.015, r = −0.612), molar proportion of acetate (*p* = 0.004, r = −0.702), and acetate:propionate (*p* = 0.03, r = −0.560). *Akkermansia* was negatively correlated with SLW (*p* = 0.004, r = −0.690), HCW (*p* = 0.002, r = −0.738), ADG (*p* = 0.007, r = −0.665), DP (*p* = 0.030, r = −0.559), and molar proportion of propionate (*p* = 0.004, r = −0.694), and positively correlated with shear force (*p* = 0.038, r = 0.540), CL (*p* = 0.029, r = 0.562), molar proportion of acetate (*p* = 0.006, r = 0.673), and acetate:propionate (*p* < 0.001, r = 0.811). *UCG_002* was negatively correlated with SLW (*p* = 0.005, r = −0.682), HCW (*p* < 0.05, r = −0.792), ADG (*p* < 0.001, r = −0.863), DP (*p* = 0.013, r = −0.625), and molar proportion of propionate (*p* = 0.001, r = −0.744), and positively correlated with shear force (*p* = 0.008, r = 0.654), DL (*p* = 0.018, r = 0.600), CL (*p* = 0.033, r = 0.552), b* value (*p* = 0.002, r = 0.729), molar proportion of acetate (*p* < 0.001, r = 0.794), and acetate:propionate (*p* = 0.003, r = 0.714). *Prevotellaceae_UCG_003* was positively correlated with a* value (*p* = 0.011, r = 0.636). *Lachnoclostridium* was positively correlated with pH_45min_ (*p* = 0.021, r = −0.589) and molar proportion of butyrate (*p* = 0.03, r = 0.558). *Alistipes* were positively correlated with a* value (*p* = 0.037, r = 0.541) and negatively correlated with the molar proportion of isobutyrate (*p* = 0.049, r = −0.515). *28_4* was positively correlated with grade rule (*p* = 0.002, r = 0.725) and molar proportion of propionate (*p* = 0.04, r = 0.536). *Rikenellaceae_RC9_gut_group* was negatively correlated with the molar proportion of valerate (*p* = 0.044, r = −0.526).

## 4. Discussion

Tibetan sheep, as a unique primitive sheep breed on the QTP, has gained much research interest from both a basic perspective and a livestock production perspective. Understanding the physiological responses of Tibetan sheep can guide management and husbandry practices in the QTP in a growing Tibetan sheep production industry. In the present study, we attempted to gain a better understanding of the Tibetan sheep gut microbiota and the effects of dietary C:F ratio on it while correlating the observed changes with slaughter performance and meat quality.

### 4.1. Effect on Slaughter Performance

Slaughter performance, as determined by carcass weight, slaughter ratio, and EMA, reflects production performance and directly impacts the amount of saleable meat, thereby affecting farmers’ income [1,21]. A high EMA value indicates better meat production performance [1]. In the present study, Tibetan sheep fed the C70 diet exhibited increased SLW, HCW, DP, EMA, and ADG compared with those in the C30 group. Additionally, animals in the C50 group showed lower HCW and EMA compared with those in the C70 group, indicating that a diet consisting of a 70:30 C:F ratio results in the optimization of slaughter performance in Tibetan sheep. The improved slaughter performance can be attributed to the lower fiber content and higher energy intake in animals receiving an increased C:F ratio in their diet, which promotes a shift in rumen fermentation from acetic acid towards propionic acid, thereby reducing the acetic acid/propionic acid ratio [22,23]. This alteration is conducive to an increase in the indicator of improved feed energy utilization efficiency [23]. This may also explain the higher EMA value observed in the C70 group. In line with the current results, Jin et al. [24] reported that lambs exhibited an increase in HCW, DP, EMA, and growth rate when dietary concentrate proportions were increased from 35% to 65%. Collectively, the findings of this research suggested that Tibetan sheep fed with a 70% concentration level for off-site fattening can lead to an improvement in slaughter performance for farmers.

### 4.2. Effect on Meat Quality Attributes

Meat quality is commonly assessed by parameters such as pH, color, tenderness, and juiciness. More specifically, an increase in pH value indicates higher fresh-keeping performance of meat [25]. In the present study, the pH_45min_ and pH_24h_ were not affected by different dietary C:F ratios, suggesting that an increased dietary C:F ratio did not change meat fresh-keeping performance.

Meat color has a direct impact on the appearance and economic value of meat [26], and an increase in the yellowness value is indicative of lower meat freshness [27]. The present study showed that increasing the dietary C:F ratio led to a significant decrease in the b* value of Tibetan sheep meat, indicating that an increase in the dietary C:F ratio significantly improved meat freshness. Increased tenderness, as indicated by reduced shear force, combined with increased juiciness, as indicated by decreased cooking loss and drip loss, likely affects the meat’s acceptability and palatability by the consumer [15,28]. The results discussed herein showed that increasing the dietary C:F ratio significantly improved meat tenderness and juiciness, which is consistent with the results of previous studies [29,30]. This variation might be explained by the fact that an increase in concentrate levels led the rumen microbiota to produce large amounts of propionic acid, thereby providing more precursor substances for fatty acid synthesis, which in turn increases intramuscular fat deposition [31,32,33,34]. Kang et al. [35] also found that high intramuscular fat content in meat is easily lost upon heating, thus increasing the CL. In addition, lambs in the C50 group exhibited L* values greater than 34 and a* values greater than 9.5, which are considered above the level acceptable by consumers [36]. Therefore, this study recommends that farmers utilize a diet with a 70:30 C:F ratio to achieve superior juiciness and tenderness in Tibetan mutton production, while a 50:50 C:F feeding diet is advised for optimal meat color.

### 4.3. Effect on Rumen Fermentation Parameters

The total VFA and VFA molar ratio plays a crucial role in evaluating the internal environmental indicators of rumen fermentation [12,24]. In the present study, an increase in C:F ratio significantly increased total VFA concentration in the rumen. This may be explained by the higher carbohydrate and starch contents in diets with a higher C:F ratio, resulting in a higher concentration of ruminal total VFA, which is consistent with findings obtained from Holstein cattle [37]. The present study also showed that the proportion of acetate and the acetate/propionate ratio decreased significantly with an increase in the C:F ratio, while the proportion of propionate increased with an increase in the C:F ratio. This may be related to the changes in rumen fermentation patterns among different C:F ratio groups. Previous studies have indicated that the abundance of cellulose-degrading bacteria in the rumen increased at higher roughage levels, leading to acetate-dominant fermentation, while the abundance of starch-degrading bacteria in the rumen was greater at higher concentrate levels, resulting in primarily propionate acid fermentation [38]. In line with our findings, Yi et al. [23] observed a significant increase in ruminal propionate concentration and a decrease in acetate concentration and acetate-propionate ratio with increasing concentrate ratio. Cui et al. [39] also suggested that increasing the molar proportion of propionate in the rumen results in improved energy efficiency, which is crucial for enhancing carcass weight. These results thus explain why the C70 group exhibited superior slaughter performance. Unfortunately, the ruminal pH was not recorded in this study, which may be a potential limitation in evaluating the possibility of induced acidosis. However, Zhang et al. [40] reported that the rumen pH value of Tibetan sheep was 5.67 when fed a diet with a 70:30 C:F ratio, which falls within the normal range (5.5–7.0) for rumen fermentation [41].

### 4.4. Effect on Rumen and Fecal Bacterial Diversity

In the present study, the results of alpha diversity and PCoA confirmed the influence of dietary C:F ratio on the structure and composition of both rumen and fecal bacterial communities in Tibetan sheep. Five samples from each group were employed for analysis in the present study, which may present a potential limitation. In fact, increasing the dietary C:F ratio not only led to significantly lower bacterial diversity but also to a dramatic change in microbial composition, which is consistent with the findings of Pang et al. [6] and Yi et al. [23] Low rumen and fecal bacterial diversity in the C70 group could be attributed to variations in dietary C:F ratio, resulting in different contents of protein and carbohydrate and energy [10,42]. Lv et al. [43] argued that feeding the higher dietary protein and energy levels significantly reduced the abundance and diversity of the ruminal microbiota of lambs. The phenomenon was consistent with the results of previous studies on the effects of dietary C:F ratio on the rumen and fecal bacterial community composition of yak [44]. However, the results were inconsistent with those of Li [45], who found that Tibetan sheep fed a diet with a 70:30 diet showed higher diversity and richness of the ruminal microbiota than those fed a 30:70 diet. This could be explained by the differences in the experimental number of sheep (5 vs. 3 animals in each group) or environmental factors in the fattening test season month (November to January vs. May to August). Notably, deep sequencing coverage (99.0%) of the rumen and fecal epimural community could provide novel and detailed insights into the impact of the dietary C:F ratio in Tibetan sheep

### 4.5. Effect on Rumen and Fecal Bacterial Community Structure

The present study comprehensively describes the composition of the rumen and fecal bacterial community in Tibetan sheep. In rumen fluid, consistent with previous studies [46,47,48], the *Firmicutes* and *Bacteroidetes* were the dominant phyla in the rumen microbiota, accounting for approximately 90% of all the bacterial phyla. However, the abundances of *Firmicutes* and *Bacteroidetes*were not affected by changes in the dietary C:F ratio, possibly due to the ability of rumen microorganisms to adapt to changing levels of dietary concentrate levels through self-regulation. We found that the relative abundance of *Verrucomicrobiota* decreased with the increase in the C:F ratio, which is supported by a study by Yi et al. [23], which reported a lower abundance of *Verrucomicrobiota* in the rumen of yaks fed with a higher C:F ratio. This change may be related to the animal’s ability to improve cellulose degradation in the rumen and conversion into VFAs for subsequent use by other organisms [49] because diets with high forage ratios feature more resistant lignocellulose. Gharechahi et al. [50] also demonstrated that *Verrucomicrobiota-*enriched genes are associated with the degradation of lignocellulosic polymers and the fermentation of degradation products into VFAs. At the genus level, only two differential genera were observed among the top 14 most abundant taxa, indicating that the majority of the genera present in the three treatment groups were not affected by the different dietary treatments, which is consistent with the results reported by Du et al. [46] Notably, the relative abundance of *Butyrivibrio* was significantly higher in the C50 group compared with the C30 group, with the C70 group exhibiting intermediate values. Consistent with the results of the present study, Petri et al. [51] found that the abundance of *Butyrivibrio fibrisolvens* in the rumen of yaks was high when animals were fed a diet composed of 30% concentrate, intermediate for a diet of 80% concentrate, and low for a diet of 0% concentrate, although the specific functions of *Butyrivibrio* remain unclear. *Ruminococcus* is one of the most important species in the rumen because it is positively correlated with carbohydrate and energy metabolism [52]. In our study, *Ruminococcus* was most abundant in the C70 group, indicating that the ability of Tibetan sheep fed to degrade starch and obtain energy from carbohydrates was improved on a diet with a 70:30 C:F ratio, which would promote the growth of Tibetan sheep.

The phyla *Firmicutes* and *Bacteroidetes* showed the highest relative abundance in the feces of Tibetan sheep, which is consistent with previous research on lambs [53], dairy cows [54], and Yak calves [55]. *Bacteroidetes* play a crucial role in the maintenance of gut homeostasis by degrading proteins and carbohydrates and by producing VFAs [54,56]. Our findings indicate that as the ratio of concentrate in the diet increases, the abundance of *Bacteroidetes* tends to decrease and then increase, with the highest abundance observed in the C70 group. This indicates that Tibetan sheep fed a 70:30 C:F ratio exhibit enhanced activity and improved utilization of both carbohydrates and protein, which is in line with results reported by Pang et al. [6] *Proteobacteria* play a significant role in biofilm formation, fermentation, and the digestion of soluble carbohydrates [57]. The present study revealed a higher relative abundance of *Proteobacteria* in the C50 group compared with the other two groups. These results are broadly consistent with those reported by Han et al. [58], who found that the abundance of *Proteobacteria* significantly increased as the proportion of dietary concentrate increased. This could be attributed to differences in diet composition, type of animal species, and location of microorganisms. The genera *Prevotella* can use starch, protein, hemicelluloses, or pectin as energy sources [10], while *Monoglobus* has been reportedly involved in dietary fiber fermentation, being also associated with healthy microbial communities [59]. *Prevotella* exhibited the highest relative abundance in the C70 group, while *Monoglobus* was found in the highest relative abundance in the C30 group. The results of this study may be explained by the highest CP content in the C70 diet and the highest fiber content in the C30 diet [60,61]. Mao et al. [62] found that the dietary protein content was highly correlated with the *Prevotella* abundance. These results suggested that, as the concentrate ratio in the diet increases, starch and protein degradation increases, while forage fiber degradation decreases, which promotes the growth of Tibetan sheep but may negatively impact host health. Notably, although the precise function of *UCG_002* is unclear, its abundance increased as the C:F ratio increased, indicating a possible role in the digestion and utilization of non-structural carbohydrates and protein.

### 4.6. Correlation Analysis

Understanding the links between the gastrointestinal bacterial community and carcass characteristics and meat quality is key to improving animal productivity and meat quality [63]. In the present study, the abundance of seven microbial genera in the rumen and seven microbial genera in the feces showed a strong association with slaughter performance and meat quality. In rumen fluid, *Saccharofermentans* and *Bacteroidales_bacterium_Bact_22* were found to be negatively correlated with the SLW or ADG. Although the causal relationship between the host and microbiota remains unclear, it can be hypothesized that these two genera are involved in lipid and protein metabolism. Moreover, *Treponema* and *Ruminococcus* were found to be positively correlated with meat color (indicated by the a* value) and juiciness (indicated by DL), whereas *Succiniclasticum* was positively correlated with meat tenderness (indicated by shear force) and juiciness (indicated by DL). These findings indicate that, to some extent, changes in meat quality may be associated with differential impacts on gut bacteria to some extent. Similar results were also observed in a previous study by Xiang et al. [63], who found that rumen bacteria composition is closely related to meat quality in sheep. In addition, four bacterial genera showed a significantly correlated with rumen VFAs of Tibetan sheep. The relative abundance of *Prevotella* was positively correlated with butyrate proportion, similar to results found by Chiquette et al. [61] and Bi et al. [64] In line with our findings, Anderson et al. [65] and Ferrario et al. [66] reported that the *Ruminococcus* was related to its ability to digest resistant starch, and its relative abundance positively correlated with valerate. This can also explain the trend towards a higher proportion of valerate in the C70 group. In addition, the abundance of *Treponema* was positively associated with the total VFA concentration, indicating the potentially pivotal role of the microbe in the microbial breakdown of dietary carbohydrates and VFA production.

In feces, the relative abundance of *Prevotella* was positively correlated with carcass traits, including SLW, ADG, HCW, and DP, but negatively correlated with shear force, cooking loss, and b* value; in contrast, *Akkermansia* and *UCG_002* were negatively correlated with carcass traits but positively correlated with meat tenderness and juiciness. These results showed that rapid ruminant growth does not necessarily correspond to better meat quality by modulating rumen bacteria. Furthermore, *Christensenellaceae_R_7_group* was negatively correlated with SLW and ADG. However, Zhao et al. [19] reported that the genus was positively correlated with ADG in lambs, which is in contrast with the findings of the present study. The reason for this discrepancy is currently unclear and warrants further investigation. Lastly, the genera *Alistipes* and *Prevotellaceae_UCG_003* were found to be positively correlated with the a* value, indicating that an increased abundance of these genera can improve meat color. *Akkermansia* can utilize intestinal mucins to produce acetate and propionate for the host’s intestinal epithelial cells [67]. The present study showed that the relative abundance of *Akkermansia* was positively correlated with the acetate proportion and negatively correlated with the propionate proportion, which could be beneficial for gastrointestinal tract health. Additionally, the propionate proportion was positively correlated with *Prevotella* and *28_4* and negatively correlated with *UCG_002* and *Akkermansia*. However, due to the complex nature of bacterial interactions, it remains challenging to pinpoint the specific bacteria responsible for producing a particular VFA. We also noticed that the abundance of *Rikenellaceae_RC9_gut_group* was negatively related to the valerate proportion, and that the same relationship exists among *Alistipes* and the level of proportion. Taken together, these findings provide a better understanding of the relationships between slaughter performance, meat quality, rumen fermentation, and microbial functions and that these relationships are influenced by dietary C:F ratios, thus potentially contributing to the development of Tibetan sheep husbandry strategies.

## 5. Conclusions

The study showed that different dietary C:F ratios could significantly influence carcass traits, meat quality, and gastrointestinal microbiota composition in Tibetan sheep. It was found that increasing the dietary concentrate level from 30% to 70% exerted a positive effect on SLW, HCW, DP, EMA, ADG, and the tenderness and juiciness of the meat of Tibetan sheep. Moreover, animals fed with 50% concentrate exhibited optimizing meat color which was more acceptable by consumers. Furthermore, correlation analysis revealed a strong correlation between specific gastrointestinal bacteria and slaughter performance, and meat quality in Tibetan sheep. However, despite limitations in rumen fermentation parameters and probiotic quantification, this study underscores the potential of C: F ratio dietary manipulation to improve both slaughter performance and meat quality in Tibetan sheep. Additionally, it elucidates the relationship between slaughter performance and gastrointestinal microbiota, providing valuable insights for farmers in selecting the appropriate C:F ratio for feeding Tibetan sheep rearing. In summary, Tibetan sheep fed with a 70:30 C:F ratio have the potential for improved slaughter performance and meat quality during cold season fattening in allopatry.

## Figures and Tables

**Table 1:** Ingredients and nutritional composition of three C:F ratio diets (DM%).

Index	Dietary Treatment ^1^		
	C30	C50	C70
Ingredients (%)			
Corn	19.50	32.50	45.50
Soybean meal	7.20	12.00	16.80
Wheat bran	1.50	2.50	3.50
Rice straw	31.50	22.50	13.50
Sweet sorghum silage	38.50	27.50	16.50
Salt	0.15	0.25	0.35
NaHCO_3_	0.45	0.75	1.05
Premix ^2^	1.20	2.00	2.80
Nutrient composition (%)			
Crude protein	11.45	13.35	15.25
Ether extract	2.40	2.56	2.72
Neutral detergent fiber	50.81	40.16	29.50
Acid detergent fiber	36.87	27.78	18.69
Calcium	0.92	0.92	0.92
Phosphorus	0.25	0.33	0.41
Metabolizable energy ^3^, MJ/kg	12.92	13.01	13.09

^1^ C30 = 30:70 concentrate: forage; C50 = 50:50 concentrate: forage; C70 = 70:30 concentrate: forage. ^2^ Formulated to provide (per kilogram of premix) 50 KIU of vitamin A, 10 KIU of vitamin D3, 450 mg of vitamin E, 2100 mg of Zn, 8 mg of Se, 900 mg of I, 2100 mg of Fe, 10 mg of Co, 30 mg of Mn, and 1 350 mg of Cu. ^3^ Calculated according to Ministry of Agriculture of P.R. China, 2004.

**Table 2:** Effects of dietary concentrate-to-forage ratios on slaughter performance of Tibetan sheep.

Index	Dietary Treatment ^1^			SEM	*p*-Value
	C30	C50	C70		
Slaughter live weight (kg)	28.20 ^b^	32.48 ^a^	34.36 ^a^	0.81	<0.001
Hot carcass weight (kg)	10.59 ^c^	13.18 ^b^	15.21 ^a^	0.54	<0.001
Dressing percentage (%)	37.64 ^b^	40.76 ^ab^	44.23 ^a^	1.02	0.016
Eye muscle area (cm^2^)	10.73 ^b^	13.36 ^b^	16.44 ^a^	0.80	0.004
Grade rule (cm)	0.81	0.79	0.96	0.03	0.068
Average daily gain (kg/d)	0.13 ^b^	0.20 ^a^	0.22 ^a^	0.01	<0.001

^a–c^ Mean values accompanied by different letters within a row indicate significant differences. ^1^ C30 = 30:70 concentrate: forage; C50 = 50:50 concentrate: forage; C70 = 70:30 concentrate: forage.

**Table 3:** Effects of dietary concentrate-to-forage ratios on meat quality attributes of Tibetan sheep.

Index	Dietary Treatment ^1^			SEM	*p*-Value
	C30	C50	C70		
pH_45min_	6.40	6.48	6.34	0.06	0.634
pH_24h_	5.46	5.52	5.52	0.02	0.414
Shear force (N)	47.91 ^a^	42.50 ^b^	26.88 ^c^	2.56	<0.001
Cooking loss (%)	40.64 ^a^	38.88 ^b^	35.67 ^c^	0.61	<0.001
Drip loss (%)	10.74 ^a^	9.77 ^a^	4.03 ^b^	0.86	<0.001
Lightness	34.57	34.40	32.74	0.51	0.280
Redness	9.27	9.61	10.44	0.23	0.101
Yellowness	6.50 ^a^	5.61 ^b^	4.61 ^c^	0.22	<0.001

^a–c^ Mean values accompanied by different letters within a row indicate significant differences. ^1^ C30 = 30:70 concentrate: forage; C50 = 50:50 concentrate: forage; C70 = 70:30 concentrate: forage.

**Table 4:** Effects of dietary concentrate-to-forage ratios on rumen fermentation parameters of Tibetan sheep.

Items	Dietary Treatment ^1^			SEM	*p*-Value
	C30	C50	C70		
Total volatile fatty acids, mmol/L	64.25 ^b^	65.00 ^b^	70.23 ^a^	1.06	0.027
Acetate (% molar)	49.98 ^a^	36.91 ^b^	24.01 ^c^	2.95	<0.001
Propionate (% molar)	24.12 ^c^	36.42 ^b^	48.69 ^a^	2.89	<0.001
Butyrate (% molar)	10.08	10.78	11.50	0.83	0.809
Isobutyrate (% molar)	4.80 ^ab^	5.03 ^a^	4.06 ^b^	0.17	0.049
Valerate (% molar)	3.78	3.58	4.49	0.18	0.074
Isovalerate (% molar)	7.24	7.30	7.25	0.22	0.995
Acetate:propionate	2.15 ^a^	1.01 ^b^	0.51 ^c^	0.20	<0.001

^a–c^ Mean values accompanied by different letters within a row indicate significant differences. ^1^ C30 = 30:70 concentrate: forage; C50 = 50:50 concentrate: forage; C70 = 70:30 concentrate: forage.

**Table 5:** Effect of dietary concentrate-to-forage ratios on alpha-diversity indices of the ruminal and fecal microbial communities in the Tibetan sheep.

Index	Dietary Treatment ^1^			SEM	*p*-Value
	C30	C50	C70		
Rumen samples					
Chao1	1579.27 ^a^	1602.15 ^ab^	1182.70 ^b^	79.27	0.026
Simpson	0.99	0.99	0.98	0.002	0.076
Shannon	8.82 ^a^	8.78 ^ab^	7.92 ^b^	0.14	0.025
PD_whole_tree	18.83	20.06	17.72	0.46	0.208
Coverage	99.98	99.98	99.99	0.002	0.131
Fecal samples					
Chao1	1456.99	1078.07	798.75	118.22	0.054
Simpson	0.99	0.98	0.98	0.004	0.222
Shannon	8.67 ^a^	8.01 ^ab^	7.47 ^b^	0.20	0.039
PD_whole_tree	15.88 ^ab^	20.51 ^a^	11.23 ^b^	1.65	0.007
Coverage	99.98	99.99	99.99	0.002	0.096

^a–b^ Mean values accompanied by different letters within a row indicate significant differences. ^1^ C30 = 30:70 concentrate: forage; C50 = 50:50 concentrate: forage; C70 = 70:30 concentrate: forage.

## Data Availability

Data used and analyzed during this study are available from the corresponding author upon reasonable request.

## Supplementary Materials

The following supporting information can be downloaded at: https://www.mdpi.com/article/10.3390/ani14223305/s1; Table S1: Comparison of similarities in microbiota composition between lambs in the three treatment groups by ANOSlM analysis; Table S2: Relative abundance of top 7 phyla in the ruminal and fecal microbiome of Tibetan sheep fed diets with different concentrate-to-forage ratios; Table S3: Relative abundance of the top 14 genera in the ruminal and fecal microbiome of lambs Tibetan sheep fed diets with different concentrate-to-forage ratios.
