# Supplementary material for: Effect of Dietary Concentrate-to-Forage Ratios During the Cold Season on Slaughter Performance, Meat Quality, Rumen Fermentation and Gut Microbiota of Tibetan Sheep
Source: Animals (Basel). 2024 Nov 17;14(22):3305. doi: 10.3390/ani14223305 (PMC11591461; doi:10.3390/ani14223305)
Supplement: Supplementary file 1 [file animals-14-03305-s001.zip › animals-3267662-supplementary.pdf]

Supplementary Table S1. Comparison of simlarities in microblota composition between lambs in the three treatment groups by ANOSlM analysis. Unweighted unifrac distance

	***R*-value**	***P*-value**
Rumen samples		
C30 *vs.* C50 [1]	0.144	0.096
C30 *vs.* C70	0.524	0.007
C50 *vs.* C70	0.260	0.020
Fecal samples		
C30 *vs.* C50 [1]	0.144	0.051
C30 *vs.* C70	0.660	0.007
C50 *vs.* C70	0.268	0.028

[1] C30 = 30:70 concentrate: forage; C50 = 50:50 concentrate: forage; C70 = 70:30 concentrate: forage.

Supplementary Table S2. Relative abundance of top 7 phyla in the ruminal and fecal microbiome of Tibetan sheep fed diets with different concentrate-to-forage ratios.

**Index**	**Dietary Treatment [1]**			**SEM**	***P*-value**	**[z]*P*-value**
	**C30**	**C50**	**C70**			
Rumen samples						
Bacteroidota	49.38	46.24	47.20	1.62	0.914	0.949
Firmicutes	41.33	45.15	41.78	1.27	0.264	0.640
Spirochaetota	1.45	1.08	4.19	0.72	0.379	0.640
Patescibacteria	1.78	2.11	1.24	0.26	0.691	0.825
Verrucomicrobiota	2.20 [a]	2.09 [a]	0.71 [b]	0.26	0.018	0.499
Actinobacteriota	1.47	0.69	0.59	0.39	0.733	0.825
Fibrobacterota	0.31	0.11	1.85	0.36	0.147	0.640
Fecal samples						
Firmicutes	53.23	41.92	51.22	3.53	0.691	0.801
Bacteroidota	31.43 [b]	18.99 [b]	40.02 [a]	2.85	0.004	0.054
Proteobacteria	2.85 [b]	15.74 [a]	2.21 [b]	2.42	0.048	0.277
Spirochaetota	5.23	4.70	3.93	0.92	0.756	0.812
Actinobacteriota	0.37	4.42	0.62	1.18	0.733	0.812
Verrucomicrobiota	2.32	1.30	0.49	0.31	0.075	0.326
Patescibacteria	0.41	1.37	0.84	0.23	0.145	0.383

[a-b] Mean values accompanied by different letters within a row indicate significant differences. [1] C30 = 30:70 concentrate: forage; C50 = 50:50 concentrate: forage; C70 = 70:30 concentrate: forage. [z]*P*-value is an adjusted BH-FDR correction value.

Supplementary Table S3. Relative abundance of the top 14 genera in the ruminal and fecal microbiome of lambs Tibetan sheep fed diets with different concentrate-to-forage ratios.

Index	Dietary Treatment [1]			SEM	*P*-value	[z]*P*-value
	C30	C50	C70			
Rumen samples						
Prevotella	10.36	8.62	9.62	0.90	0.827	0.908
Rikenellaceae_RC9_gut_group	8.95	8.07	6.43	0.57	0.357	0.553
Christensenellaceae_R_7_group	5.87	6.26	6.31	0.63	0.932	0.974
Succiniclasticum	4.26	3.46	6.02	0.45	0.090	0.553
NK4A214_group	3.56	4.11	3.88	0.44	0.932	0.974
Prevotellaceae_UCG_003	2.11	2.38	2.97	0.35	0.613	0.731
Treponema	1.39	1.05	4.16	0.71	0.379	0.568
Butyrivibrio	0.78[b]	3.08[a]	1.46[ab]	0.38	0.031	0.481
Prevotellaceae_UCG_001	1.78	1.72	1.68	0.24	0.914	0.963
Saccharofermentans	2.12	1.29	1.53	0.16	0.121	0.553
Candidatus_Saccharimonas	1.15	1.83	0.86	0.26	0.512	0.710
Ruminococcus	0.91[ab]	0.71[b]	1.60[a]	0.15	0.042	0.510
Veillonellaceae_UCG_001	0.59	1.43	1.01	0.18	0.310	0.553
Bacteroidales_bacterium_Bact_22	1.80	0.38	0.49	0.32	0.196	0.553
Fecal samples						
UCG_005	5.40	5.61	6.35	0.70	0.811	0.848
Rikenellaceae_RC9_gut_group	7.19	3.62	5.08	0.85	0.068	0.448
Alistipes	4.34	2.93	6.04	0.76	0.481	0.566
Treponema	4.65	4.61	3.93	0.90	0.914	0.931
Christensenellaceae_R_7_group	5.83	3.77	3.41	0.53	0.249	0.448
Bacteroides	4.28	2.04	5.05	0.52	0.065	0.448
Succinivibrio	2.32	6.44	0.44	1.71	0.686	0.738
Prevotellaceae_UCG_003	2.90	1.43	2.49	0.80	0.402	0.485
Monoglobus	3.24 [a]	0.77 [b]	2.33 [ab]	0.49	0.018	0.361
Lachnoclostridium	1.72	2.16	1.42	0.24	0.330	0.448
Prevotella	0.20 [b]	0.32 [b]	4.00 [a]	0.61	0.013	0.302
28_4	1.10	1.17	2.24	0.31	0.454	0.539
UCG_002	2.57 [a]	1.08 [b]	0.62 [b]	0.28	0.013	0.302
Akkermansia	1.84	0.67	0.44	0.27	0.089	0.448

[a-b] Mean values accompanied by different letters within a row indicate significant differences. [1] C30 = 30:70 concentrate: forage; C50 = 50:50 concentrate: forage; C70 = 70:30 concentrate: forage. [z]*P*-value is an adjusted BH-FDR correction value.
